# Elevation of SHANK3 Levels by Antisense Oligonucleotides Directed Against the 3′-UTR of the Human *SHANK3* mRNA

**DOI:** 10.1089/nat.2022.0048

**Published:** 2023-02-01

**Authors:** Nadine Stirmlinger, Jan Philip Delling, Stefanie Pfänder, Tobias M. Boeckers

**Affiliations:** ^1^ Institute of Anatomy and Cell Biology and Ulm University, Ulm, Germany.; ^2^ International Graduate School for Molecular Medicine, Ulm University, Ulm, Germany.; ^3^ DZNE, Ulm Site, Ulm, Germany.

**Keywords:** ASOs, PMDS, SHANK3, mRNA stabilization, hiPSC

## Abstract

SHANK3 is a member of the SHANK family of scaffolding proteins that localize to the postsynaptic density of excitatory synapses. Mutations within the *SHANK3* gene or *SHANK3* haploinsufficiency is thought to be one of the major causes for Phelan-McDermid Syndrome (PMDS) that is characterized by a broad spectrum of autism-related behavioral alterations. Several approaches have already been proposed to elevate SHANK3 protein levels in PMDS patients like transcriptional activation or inhibition of SHANK3 degradation. We undertook a systematic screening approach and tested whether defined antisense oligonucleotides (ASOs) directed against the 3′ untranslated region (3′-UTR) of the human *SHANK3* mRNA are suitable to elevate SHANK3 protein levels. Using human induced pluripotent stem cells (hiPSCs) and hiPSCs-derived motoneurons from controls and PMDS patients we eventually identified two 18 nucleotide ASOs (ASO 4-5.2-4 and 4-5.2-6) that were able to increase SHANK3 protein levels *in vitro* by about 1.3- to 1.6-fold. These findings were confirmed by co-transfection of the identified ASOs with a GFP-SHANK3-3′-UTR construct in HEK293T cells using GFP protein expression as read-out. Based on these results we propose a novel approach to elevate SHANK3 protein concentrations by 3′-UTR specific ASOs. Further research is needed to test the suitability of *SHANK3*-specific ASOs as pharmacological compounds also *in vivo*.

## Introduction

SHANK3 is a major synaptic scaffolding protein of the postsynaptic density (PSD) of excitatory synapses that plays an important role in the formation and maturation of synapses and dendritic spines [1–4]. *SHANK3* haploinsuffiency on account of the heterozygous loss of the distal arm of chromosome 22 or to mutations within the *SHANK3* gene [5] leads to a syndromic form of a neurodevelopmental autism spectrum disorder (ASD) named 22q13.3 deletion or Phelan-McDermid Syndrome (PMDS) [6–8]. The syndrome has a wide variety of symptoms and is mainly characterized by autism-related behaviors [5,9] in addition to neonatal hypotonia, global developmental and speech delay, and mild to severe intellectual disability. Haploinsuffiency of *SHANK3* in PMDS patients leads to a diminished expression of *SHANK3* mRNA and transcribed protein isoforms to about 50%–80% compared to controls in human induced pluripotent stem cell (hiPSC)-derived neurons from PMDS patients [10,11].

Based on these findings there have been several *in vitro* and *in vivo* screening approaches to find compounds to restore neuronal deficits and/or behavioral abnormalities by elevating SHANK3 levels [10,12]. Compounds showing a positive effect on excitatory synaptic activity or behavioral aspects are IGF-1, CLK2 inhibitors, oxytocin or Lithium [10,11,13–18]. Lithium has already successfully been applied to PMDS patients and beneficial effects on behavior have been reported [19,20]. Recently, NNZ-2591 has been tested in *Shank3* knock-out mice and was shown to restore social interaction, repetitive behavior, and motor deficits. Additionally, a phase I clinical trial has been completed in PMDS patients [21].

Although these treatment strategies are being tested, further options to target specifically *SHANK3* are needed. Currently, the first investigational gene replacement therapy for *SHANK3* has reached the preclinical stage. JAG201 is designed to correct genetic function of *SHANK3* via an AAV9 vector [22]. Considering their high target specificity, the identification and application of antisense oligonucleotides (ASOs) poses another promising alternative [23]. ASOs are synthetic single stranded strings of nucleic acids that are complementary to a gene's sequence or parts of it. ASOs can bind by Watson-Crick hybridization to the mRNA transcript of their complementary sequence and hence can influence splicing, silence or promote the expression of a target protein [23]. Extensive research has been performed to improve pharmacokinetic properties of ASOs by modifying the backbone or sugar groups yielding increased stability and uptake and to determine the optimal length, which appears to be in the range of 16 to 20 base pairs [24,25].

In the past years, interest in ASO based approaches has peaked when Nusinersen/Spinraza, an ASO drug based on the principle of exon inclusion for spinal muscular atrophy (SMA) [26], was approved globally [27–30]. ASOs for other neurodegenerative disorders are currently in different stages of clinical trials [21,23].

The majority of ASOs lower transcript levels of proteins, usually due to RNase H mediated degradation or steric hindrance. However, it is also possible to increase the amount of transcribed protein by mRNA stabilization or splicing interference [23,31]. The role of the 3′ untranslated region (UTR) in mRNA stability has been investigated extensively in the past and revealed an association of AU-rich sequences to rapid RNA degradation [32,33]. Recently, Li *et al*. could show that targeting the 3′ or the 5′-UTR with ASOs could stabilize *Frataxin* mRNA and thereby increase Frataxin protein levels [34]. This finding proves that targeting sequences within the 3′-UTR to impede rapid degradation poses a promising approach toward increasing mRNA stability and by this enhancing mRNA translation. Quite interestingly, past studies showed that certain miRNAs have an effect on the post-transcriptional regulation of *SHANK3* expression and that these miRNAs bind to the *SHANK3* 3′-UTR downregulating expression levels [35,36].

All these studies prompted us to screen for ASOs directed against the 3′-UTR that might be able to increase SHANK3 expression. In a first step we investigated the effect of 50 non-overlapping nucleotide (nt) long ASOs directed against the complete length of the 3′-UTR of the *SHANK3* mRNA. ASO transfected human iPSCs and hiPSC-derived motoneurons from controls and PMDS patients were subsequently analyzed for SHANK3 protein expression. We found four 50nt ASOs that were able to significantly increase the protein levels of SHANK3. Further analysis of these target sequences was done by 18nt ASOs that were extensively tested in control (CTRL) and PMDS hiPSCs and hiPSC-derived motoneurons. Two ASOs were eventually identified that enhanced SHANK3 expression by about 1.3–1.6-fold.

## Materials and Methods

### hiPSCs and differentiation

hiPSCs were generated from keratinocytes of PMDS patients and healthy controls a previously established protocol [37] to reprogram them into hiPSCs. Two healthy control cell lines and three cell lines generated from PMDS patients were used [11] (Supplementary Table S1). hiPSCs were cultured on plates coated with Matrigel (Corning, New York, USA) in mTeSR1 medium (StemCell Technologies, Vancouver, Canada). At a confluence of ∼80%, cells were split in a ratio of 1:3 to 1:8 using Dispase (StemCell Technologies) and transferred to a new six-well plate.

***Motoneurons*** were generated from hiPSCs. Motoneurons used in the experiments for Figs. 2 and 3D were generated following the protocol published by Hu and Zhang [38] and Reinhardt *et al*. [39]. Motoneurons used for experiments are displayed in Fig. 3A–C, we followed the protocol as published by Catanese *et al.* [40].

**FIG. 1.**
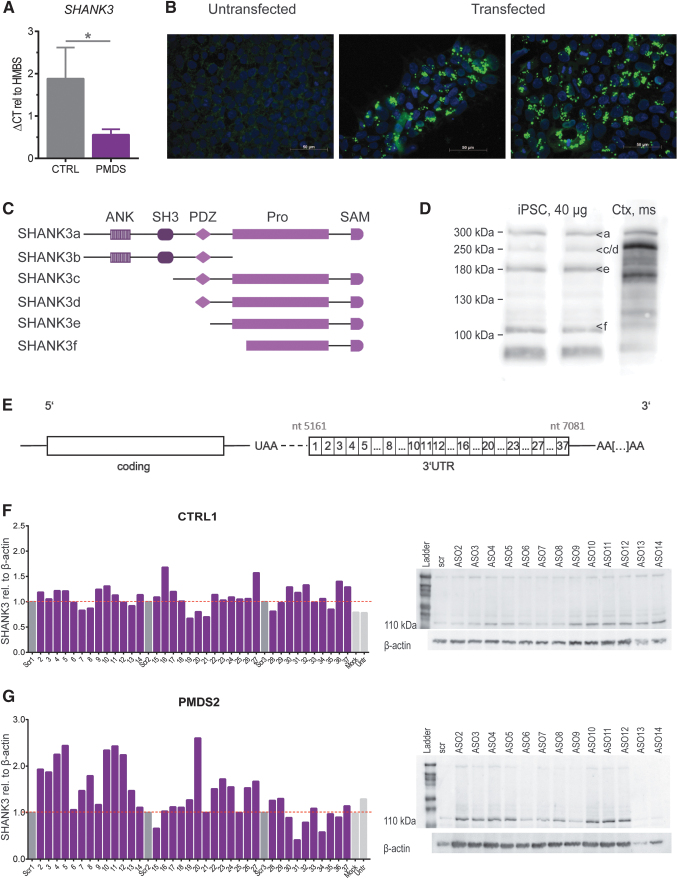
Transfection of human CTRL and PMDS hiPSCs with 50 bp ASOs. **(A)** SHANK3 mRNA levels in CTRL versus PMDS patients (*n* = 3, bars represent mean + SEM). **(B)** Transfection efficiency is visualized by biotin tagged scr ASO, DAPI in *blue*, biotin in *green*. **(C)** Schematic view on SHANK3 isoforms coding for different protein–protein interaction domains. **(D)** Exemplary western blot showing the pattern of the major SHANK3 isoforms in hiPSCs and as comparison in mouse cortex (ctx, ms). **(E)** Structure of the SHANK3 mRNA indicating the position of generated ASOs (50mers, non-overlapping) covering the 3*′*-UTR of Shank3. **(F, G)** SHANK3 protein levels of the 110 kDa isoform (highly expressed in hiPSCs) after transfection were detected via western blotting and normalized on the SHANK3 levels in scrambled transfected cells. Screening for changes in SHANK3 levels of transfected CTRL1/PMDS2 hiPSCs as detected in western blot (shown ASOs 2 to 14). Some ASOs increase the SHANK3 expression whereas others decrease it compared to scrambled transfected samples. Blots for ASOs 15–37 can be found in the Supplementary Fig. S1 (*n* = 1, bars represent obtained value for the 110 kDa isoform). ASOs, antisense oligonucleotides; CTRL, control; DAPI, 4′,6-diamidino-2-phenylindole; hiPSCs, human induced pluripotent stem cells; PMDS, Phelan-McDermid Syndrome; SEM, standard error of the mean; UTR, untranslated region.

**FIG. 2.**
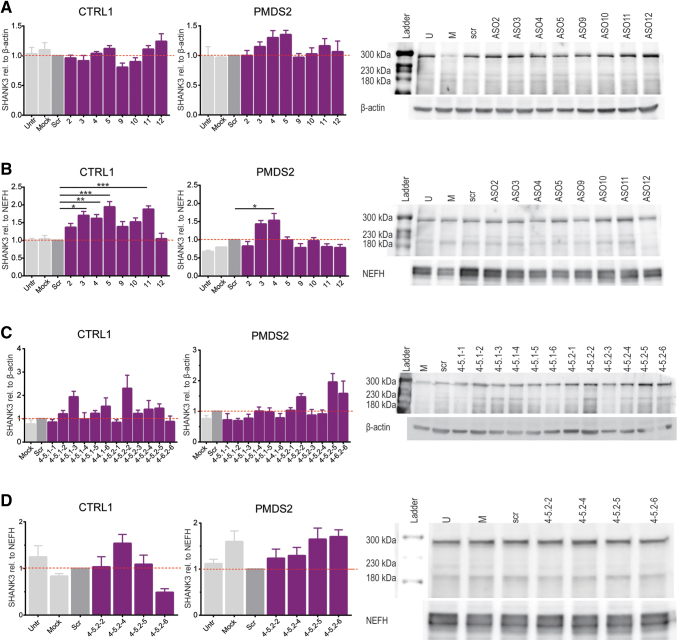
SHANK3 expression in transfected hiPSCs and motoneurons using the effective 50nt ASOs and screening for the effect of ASO 4 and ASO 5 derived 18nt ASOs. Effective ASOs have been re-evaluated in **(A)** triplicates of hiPSCs and **(B)** triplicates of human motoneurons. The transfection of motoneurons indicated that the selected ASOs have an even stronger effect in differentiated cells compared to hiPSCs. Bars represent overall SHANK3 expression (300, 230, and 180 kDa) as mean of triplicates + SEM (one-way ANOVA with *P < 0.05, **P = 0.01, ***P = 0.001). **(C)** Screening for the effect of short ASO 4 and ASO 5 derived ASOs (18mers) on the overall SHANK3 expression. Effective 18mer ASOs were more extensively tested in motoneurons from one control and one patient cell line **(D)** (300, 230, and 180 kDa) + SEM (one-way ANOVA with *P < 0.05, **P = 0.01, ***P = 0.001). ANOVA, analysis of variance.

**FIG. 3.**
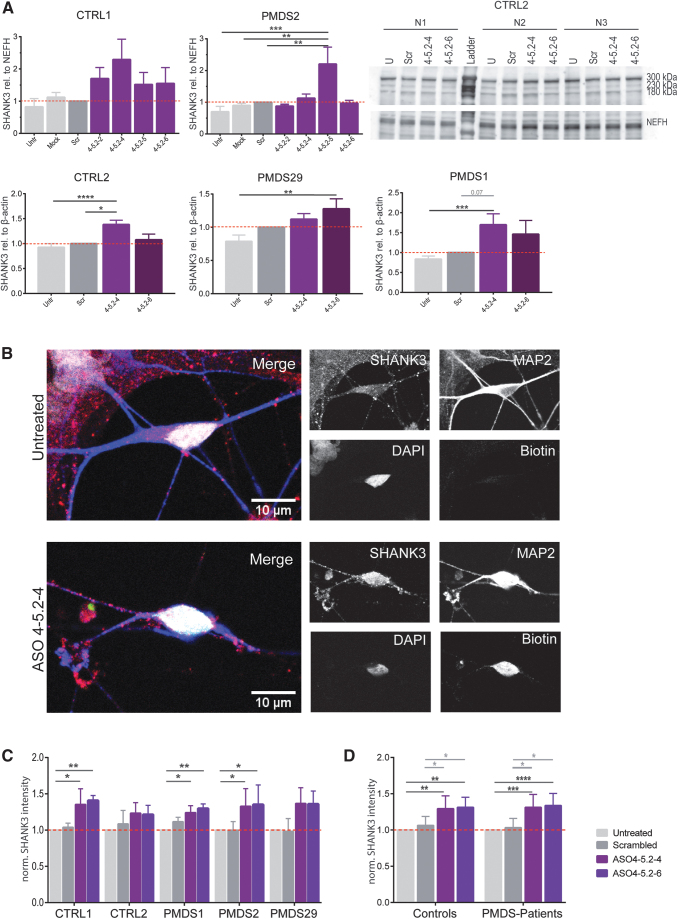
Assessment of SHANK3 levels in several independent control and PMDS hiPSC-derived motoneurons and protein detection after transfection of biotinylated 18nt ASOs using confocal imaging. **(A)** The most effective ASOs were tested in additional hiPSC cell lines from controls and PMDS patients. The CTRL1- and PMDS2-line were again transfected and analyzed as internal control. SHANK3 levels as detected by western blotting. Bars represent overall SHANK3 expression as mean of triplicates (300, 230, and 180 kDa) + SEM (one-way ANOVA with *P < 0.05, **P = 0.01, ***P = 0.001, ****P = 0.00001). **(B)** Single transfected motoneurons stained for MAP2 (*blue*), SHANK3 (*red*), Biotin (*green*), and DAPI (*white*). **(C, D)** SHANK3 intensity (mean *gray* value) was measured in the soma and normalized on the detected SHANK3 intensity in untreated motoneurons. Bars represent mean + SEM (one-way ANOVA with *P < 0.05, **P ≤ 0.01, ***P ≤ 0.001, ****P ≤ 0.0001). The ASO 4 derived 18mers used are color coded.

### Transfection of hiPSCs/motoneurons

Transfection with Lipofectamine 3000 (ThermoFisher Scientific, Waltham, Massachusetts, USA) was executed according to the manufacturers' protocol. Volumes for 12-well plates or μ-dishes: 62.5 μL DMEM/F-12 + Glutamax (Gibco, 31330-028) per tube, 0.94 μL (0.71%) LF3000, 5 μL (7.2%) P3000 and 0.5 μg ASO. Transfection for western blotting was performed in 12-well plates, motoneurons for immunocytochemistry were transfected in μ-dishes.

Transfection using the EV Shuttle Transfection Kit (BioCat; EVS110A-1-SB) was performed according to the manufacturers' instructions. Cells were incubated for 72 h and then collected for western blotting.

### HEK293T cell culture

HEK293T were cultured in flasks at 37°C and 5% CO_2_ and maintained in 10 mL DMEM with 10% fetal bovine serum (FBS). Twice a week, HEKs were split in a ratio of 1:10. For transfections, HEK293T were split into 12-well plates in a ratio corresponding to the desired culture time. Transfection times were chosen according to previously published data [34,41].

### Transformation

An electro-competent XL-1 strain of *Escherichia coli* was transformed using electroporation. One microliter of plasmid DNA and 50 μL of the cell/DNA suspension was added to a pre-cooled cuvette. After one pulse at 1,800 V, cells were resuspended in 1 mL Super Optimal Broth medium (Carl Roth, Karlsruhe, Germany) and transferred into a 2 mL reaction tube. The next day colonies were picked and resuspended in Lennox L Broth Base, Invitrogen medium supplemented with 1:1,000 Amp (LB-Amp). Glycerol stocks were prepared.

### Midi Prep

Midi Prep was performed according to the manufacturers' instructions using the NucleoBond Xtra Midi EF Kit (No. 740420.50; Macherey-Nagel, Düren, Germany). The final DNA pellet was reconstituted in endotoxin free water.

### Transfection of HEK293T cells

HEK293T cells were transfected using Polyethylenimine Max (PEI Max). Per well of a 12-well plate, 49.6 μL of DMEM were mixed with 4.5 μL of PEI Max, 1.5 μg of ASO, and/or 1.5 μg of DNA (3′ ms or 3′ hs). Plates were screened for successful transfection under the Personal AUtomated Lab Assistant (PAULA; Leica, Wetzlar, Germany). Transfected cells were collected after 24, 48, 72, or 96 h.

### Antisense oligonucleotides

DNA ASOs were produced by Eurofins (Luxemburg) and all ASOs carried three phosphorothioate modified backbones to increase stability. ASOs used for immunocytochemistry were labeled with a biotin tag at the 3′ and 5′ end to allow detection. RNA ASOs were produced by Eurogentec (Belgium). RNA ASOs consist of 2′-O-methoxyethyl bases linked by a phosphorothioate backbone. Additionally, all cytosines were 5-methyl deoxycytosines (for sequences see Supplementary Table S2).

### Immunocytochemistry

Cells were fixed for 10 min at room temperature (RT) using 4% paraformaldehyde with 0.1% sucrose (both Carl Roth). Blocking was performed with 5% FBS (Life Technologies, Carlsbad, California, USA) in PBS+/+ for >1 h. Primary antibodies (1:500 SHANK3 Tier 2 Fragment 1 + 2 α-rabbit, in house^11^; 1:1,000 Map2 α-chicken, EnCor, Gainesville, Florida, USA; 1:1,000 Biotin α-mouse, ThermoFisher Scientific) diluted in blocking solution were incubated overnight at 4°C. After application of the secondary antibodies (Jackson ImmunoResearch, West Grove, Pennsylvania, USA) slides were mounted with ProLong^®^ Gold Antifade Mountant with 4′,6-diamidino-2-phenylindole (Life Technologies).

### Western blot

Lysis using radioimmunoprecipitation assay buffer was performed on ice for 15 min. Cells were homogenized and the protein-containing supernatant was transferred to a new reaction tube and Bradford Assay was performed. Equal amount of protein was separated by sodium dodecylsulfate polyacrylamide gel electrophoresis page and transferred to a polyvinylidene fluoride membrane.

The membrane was blocked and then incubated with the primary antibodies (1:500 SHANK3 Tier 2 Fragment 1 + 2 α-rabbit, in house^11^ or 1:5,000 GFP, Santa Cruz Biotechnologies, California, USA) overnight. The horse reddish peroxidase conjugated secondary antibody was incubated for 1 h at RT and bands were detected using the MicroChemi 4.2 imager and analyzed using the GelAnalyzer or the Image Lab Software. Detected SHANK3 isoforms were the 300, 230, 180, and 110 kDa isoforms. In the initial 50nt ASO screen, the SHANK3 110 kDa isoform was analyzed due to its predominant expression and detectability in undifferentiated hiPSCs. For all other experiments, the 300, 230 and 180 kDa bands were analyzed. Used antibodies were β-actin (1:250,000, α-mouse; Sigma), Neurofilament Heavy Chain (NEFH, 1:1,000, α-chicken; Abcam), SHANK3 Tier 2 Fragment 1 + 2 (1:500, α-rabbit; Boeckers Lab) and GFP (1:5,000, α-mouse; Santa Cruz Biotechnologies).

### RNA isolation and qRT-PCR

All steps were performed at RT using the RNeasy Mini Kit (Qiagen, Venlo, Netherlands) according to the manufacturers' instructions and RNA concentration was determined using the NanoDrop. All samples were diluted to 100 ng/μL. RNA was kept on ice and sample preparation and quantitative real time polymerase chain reaction (qRT-PCR) were executed in accordance with the instructions manual (Rotor-Gene™ SYBR^®^ Green RT-PCR Kit; Qiagen). Primers were purchased from Qiagen (Hs_SHANK3_1_SG QuantiTect Primer Assay, HS_HMBS Qiagen QuantiTect Primer Assay).

### RNA secondary structure

Secondary structure of the human *SHANK3* 3′-UTR was generated using RNAfold web server.

### Data analysis and statistics

Statistical analysis was performed using GraphPad Prism7. Data were tested for normal distribution (Shapiro-Wilk test) and for homogeneity of variances (Levene test). Since more than two groups were compared, the analysis of variance (ANOVA) was performed for normally distributed data that presented homogeneity of variance. ANOVA was followed by the Tukey *post hoc* test. For non-normally distributed data, the Kruskal–Wallis test followed by a *post hoc* test was performed.

Data were shown in bars representing the mean and the standard error of the mean. Statistical significances were represented by asterisks according to their calculated probability (*P*-value). For *P* < 0.05, data were considered statistically significant and were indicated with one asterisk (*), for *P* ≤ 0.01 the significant difference was represented by two asterisks (**) and for *P* ≤ 0.001 it was considered statistically highly significant (***).

## Results

### *SHANK3* expression in hiPSCs and transfection using Lipofectamine 3000

We could confirm the *SHANK3* haploinsufficiency in PMDS-patients by detecting *SHANK3* mRNA levels in undifferentiated hiPSCs. PMDS-patients presented with a 50% reduction in mRNA amount (Fig. 1A). Next, we established the concentrations for successfully transfecting hiPSCs using Lipofectamine 3000 (Fig. 1B). Then, we performed western blots on hiPSC lysates to optimize detection of all isoforms. The *SHANK3* gene codes for a number of isoforms containing different protein–protein interaction domains (Fig. 1C). By loading at least 30 μg of protein and using pre-cast gradient gels we were able to clearly separate and detect SHANK3a (300 kDa), SHANK3 c/d (230 kDa), SHANK3e (180 kDa), and SHANK3f (110 kDa) in iPSCs (Fig. 1D). SHANK3b cannot be detected by our antibody. The SHANK3 3′-UTR was divided into thirty-seven 50nt long non-overlapping stretches, ASOs (DNA) were designed accordingly and used for the initial screen (Fig. 1E).

### Modulation of SHANK3 levels by 50nt ASOs covering the complete 3′-UTR of the *SHANK3* mRNA

SHANK3 protein expression (110 kDa SHANK3 isoform was analyzed due to its predominant expression and detectability in undifferentiated hiPSCs) was detected after transfecting hiPSCs with ASOs directed against the 3′-UTR of the *SHANK3* mRNA (Fig. 1F, G). Untransfected, mock transfected or cells transfected with a scrambled ASO were used as controls. These experiments revealed that most ASOs have no significant effect on SHANK3 protein levels; however, some ASOs obviously modulated the SHANK3 protein amounts.

50nt ASOs that caused an increase in SHANK3 levels were either clustering (ASOs 2 to 5, ASOs 9 to 12) or isolated (ASO 16, 20 and 27). More pronounced effects were seen in PMDS hiPSCs where ASOs 2 to 5 lead to a 2.1-fold increase (vs. 1.2-fold in CTRL iPSCs). ASOs 10 to 12 induced a 2.3-fold upregulation in PMDS patient cells and ASOs 9 to 11 increased SHANK3 expression 1.2-fold in controls (Fig. 1F, G). In both cell lines, other single ASOs led to an increase in protein expression, for example, ASO 16 (1.7-fold), ASO 27 (1.6-fold), or ASOs 36 and 37 (1.4-fold and 1.3-fold) in CTRL iPSCs and ASO 20 (2.6-fold) in PMDS hiPSCs (Fig. 1F, G).

Subsequently, we retested in triplicate the 50nt ASOs that were clustering at the 5′-UTR (ASOs 2 to 5; ASOs 9 to 12) in hiPSCs and hiPSCs-derived motoneurons (Fig. 2A, B). When applied to hiPSCs, the candidate ASOs chosen from the initial screening yielded an increase of about 1.2 to 1.3-fold in PMDS (ASOs 3 to 5) and in CTRL, ASOs 11 and 12 increased detected protein levels by 1.1-fold and 1.2-fold (Fig. 2A). In hiPSCs of both cell lines, ASOs 9 and 10 did not lead to higher SHANK3 levels. The same held true for ASO 3 in CTRL hiPSCs (Fig. 2A). In motoneurons, we observed a stronger effect on SHANK3 upregulation. CTLR motoneurons transfected with ASOs 2 to 5 significantly increased SHANK3 by 1.4 to 1.9-fold and CTRL motoneurons transfected with ASO 11 and increased protein levels 1.9-fold (Fig. 2B). In PMDS motoneurons, significantly more SHANK3 protein expression was detected when ASO 4 was applied (Fig. 2B).

When summarizing the different approaches, the adjacent 50nt ASOs 4 and 5 were found to be the most effective ones (Table 1) and therefore their combined sequences were chosen as the basis for the generation of smaller non-overlapping 18nt ASOs (sequences can be found in Supplementary Table S2).

**Table 1.** Summary of the Antisense Oligonucleotides that Enhance SHANK3 Protein Expression and the Cell Lines Used

	CTRL1	PMDS2	CTRL2	PMDS1	PMDS29	Overall
**50nt ASOs**						
(WB data)						
ASO 2	1.3	1.2	–	–	–	1.25
ASO 3	1.2	1.4	–	–	–	1.30
ASO 4	1.3	1.6	–	–	–	1.45
ASO 5	1.4	1.4	–	–	–	1.40
ASO 9	1.2	1.0	–	–	–	1.10
ASO 10	1.2	1.4	–	–	–	1.30
ASO 11	1.4	1.4	–	–	–	1.40
ASO 12	1.1	1.3	–	–	–	1.20
**18nt ASOs**						
(WB data)						
ASO 4-5.2-2	1.7	1.2	–	–	–	1.45
ASO 4-5.2-4	1.7	1.1	1.4	1.7	1.1	1.40
ASO 4-5.2-5	1.4	1.9	–	–	–	1.65
ASO 4-5.2-6	1.0	1.4	1.1	1.5	1.3	1.26
(IF data)						
ASO 4-5.2-4	1.35	1.23	1.24	1.33	1.37	1.30
ASO 4-5.2-6	1.41	1.21	1.30	1.35	1.36	1.33

Fold-increase of SHANK3 levels per ASO as detected per cell line. In the last column the overall effect has been calculated.

ASOs, antisense oligonucleotides; IF, immuno-fluorescence; WB, western blot.

### Modulation of SHANK3 levels by 18nt ASOs derived from the 50nt ASOs 4 and 5

When transfected with 18nt ASOs, CTRL hiPSCs showed a strong increase in SHANK3 levels followed by the treatment with ASO 4-5.1-3 (1.9-fold) and ASO 4-5.2-2 (2.3-fold). Also, the ASOs 4-5.2-3 to 4-5.2-5 led to elevated SHANK3 levels (between 1.2 and 1.4-fold). In PMDS2 hiPSCs, only the ASOs 4-5.2-2, 4-5.2-5, and 4-5.2-6 led to higher protein levels, increasing the mean protein expression by about 1.5 to 2-fold (Fig. 2C). Therefore, the ASOs 4-5.2-2, 4-5.2-4 to 4-5.2-6 were further tested in hiPSCs-derived motoneurons from CTRL1 and PMDS2. In CTRL1 motoneurons, the ASO 4-5.2-4 was the only one showing a strong effect on SHANK3 levels leading to a 1.5-fold increase (Fig. 2D). In PMDS2 motoneurons, all four tested ASOs led to higher protein levels, in average, increasing protein levels between 1.2 and 1.7-fold (Fig. 2D).

Motoneurons from more hiPSC cell lines were transfected with the most effective ASOs and again tested by western blotting (Fig. 3A). In both control lines, ASO 4-5.2-4 lead to a 1.4 to 2.3-fold increase in SHANK3 levels, whereas ASO 4.5-2-6 led to 1.1 to 1.5-fold more protein. In PMDS2, the protein level only increased after treating with ASO 4-5.2-5 (2.2-fold) and ASO 4-5.2-4 (1.1-fold). In PMDS1 and PMDS29 motoneurons, treatment with the ASOs 4-5.2-4 and 4-5.2-6 also led to higher amounts of protein (1.1 to 1.5-fold and 1.3 to 1.7-fold). Transfection with the ASO 4-5.2-4 resulted in significantly higher amounts of detected SHANK3 in CTRL2 (1.4-fold) and in PMDS1 (1.7-fold) whereas transfection with ASO 4-5.2-6 significantly increased protein levels in PMDS29 (1.3-fold) (Fig. 3D).

To analyze SHANK3 expression on a cellular level, motoneurons were transfected with biotinylated versions of the two most effective ASOs and the mean intensity of the expressed SHANK3 in the somata of transfected neurons was analyzed (Fig. 3B). Mean gray values were normalized to the SHANK3 expression of untreated cells for each cell line. SHANK3 intensity was higher when the cells were transfected with the ASOs 4-5.2-4 and 4-5.2-6 but not when transfected with the scrambled ASO (Fig. 3C). This increase became significant for both tested ASOs in CTRL1, PMDS1, and PMDS2 hiPSC-derived motoneurons (Fig. 3C). When data from controls and patients were pooled (Fig. 3D), both ASOs lead to a highly significant increase in SHANK3 intensity in both control and patient cells. ASO 4-5.2-4 augmented detected SHANK3 intensity 1.3-fold in controls and in patients. The same was observed in ASO 4-5.2-6 transfected motoneurons. Notably, the intensity of SHANK3 was also significantly higher when compared to the scrambled transfected ASOs (Fig. 3D).

### Expression of constructs containing a hybrid GFP-SHANK3 3′-UTR in HEK293T cells and analysis of GFP expression after co-expression of the identified ASOs

**FIG. 4.**
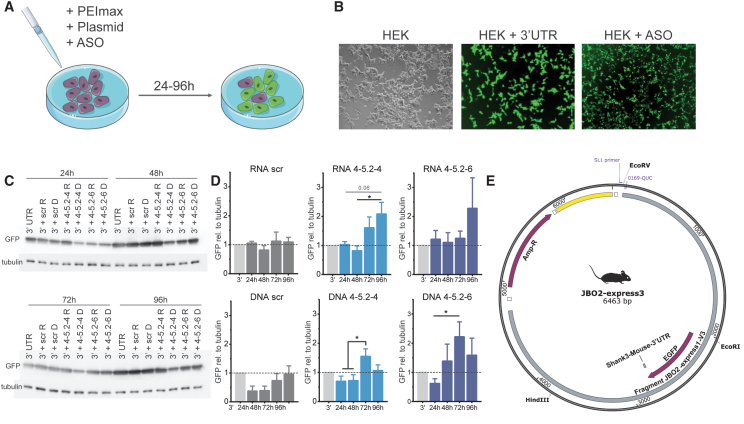

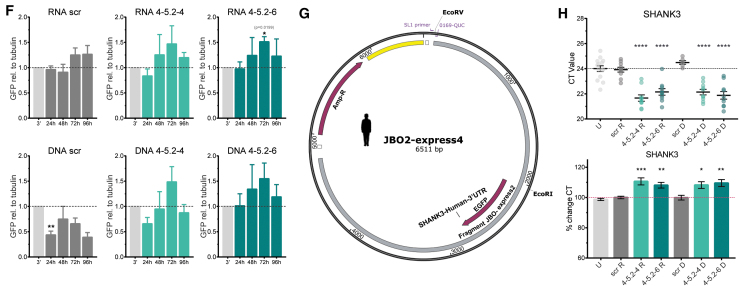
Assessment of GFP expression in transfected HEK293T cells using identified ASOs and a plasmid coding for GFP attached to the human or mouse SHANK3 3′-UTR. **(A)** Schematic overview on the transfection procedure. **(B)** Successful transfection was determined via fluorescence tagged ASOs or via GFP expression. **(C)** Western blot of transfected HEK293 cells expressing GFP after transfection and **(D)** assessment of GFP levels upon transfection with ASO 4 derived effective RNA and DNA ASOs (18mers) using the plasmid expressing GFP attached to the mouse 3′-UTR (ANOVA, *n* = 5). **(E)** Simplified vector chart of the mouse 3′-UTR construct used, a detailed vector chart can be found in the Supplementary Fig. S4. **(F)** GFP levels in HEK293 cells after co-transfection of GFP attached to the human 3′-UTR and the RNA or DNA ASO 4 derived 18mer ASOs (ANOVA, *n* = 5). **(G)** A simplified vector chart for the GFP-human SHANK3-3′-UTR plasmid; a detailed vector chart can be found in the Supplementary Fig. S2. **(H)** Endogenous SHANK3 mRNA expressed in HEK293 cells was analyzed after transfection with ASO 4 derived RNA and DNA ASOs (18mers). CT values as detected in quantitative real time polymerase chain reaction and calculated changes of CT values in percent (ANOVA, *n* = 6–11). Bars represent mean + SEM.

To confirm the effect of our ASOs in an independent, easier and more consistent system, we designed expression constructs in which we fused GFP to the Shank3 3′-UTR of the human (3′hs) or the mouse (3′mm) gene (Fig. 4E, G). Full sized vector charts along with sequencing results of the obtained DNA constructs are provided in the supplements (Supplementary Figs. S2–S5). We transfected HEK293T cells with the GFP-3′hs and GFP-3′mm constructs and/or tagged ASOs and visualized the transfection efficiency (Fig. 4A). Successful transfection was assessed using the PAULA imager (Fig. 4B). Next, we transfected HEK293T cells with the GFP-3′hs or GFP-3′mm constructs and co-transfected the identified ASOs.

We also generated RNA ASOs from our candidate ASOs the scrambled ASO, further referred to as RNA scr or DNA scr and the same nomenclature for the other ASOs to compare these two ASO versions. After transfection, we assessed the amount of expressed GFP via western blotting (Fig. 4C) after 24, 48, 72, and 96 h. Upon transfection with the GFP-3′mm construct and the scr ASOs, we observed no changes in GFP expression (Fig. 4D). Transfection with the GFP-3′mm construct and RNA 4-5.2-4 significantly increased GFP protein amount by 2.1-fold as detected after 48 h versus 96 h of transfection. We also found a trend (*P* = 0.057) in GFP increase between 24 and 96 h of transfection. After 72 h of transfection, we already observed a tendency for higher protein expression with a 1.6-fold higher GFP detected compared to the GFP-3′mm only transfected HEK293T cells (Fig. 4D).

Co-transfection of GFP-3′mm with RNA 4-5.2-6 did not show significant changes, however, at all examined time points, detected GFP was 1.1 to 2.3-fold higher than in GFP-3′mm only transfected cells (Fig. 4D). Upon transfection of HEK293T cells with GFP-3′mm and DNA ASOs, we found a significant increase in cells treated with DNA 4-5.2-4 ASO after 72 h compared to the earlier time points and in cells treated with DNA 4-5.2-6 after 72 h (Fig. 4D). When transfected with the GFP-3′hs construct and the different ASOs, we found a significant increase in GFP levels (1.5-fold) for RNA 4-5.2-6 transfected HEK293T cells after 72 h (Fig. 4F). The other RNA and DNA ASOs showed tendencies to increase GFP protein levels after 72 h of transfection by 1.4 to 1.5-fold (Fig. 4F).

The RNA scr ASO did not affect GFP expression, however, the DNA scr ASO led to significantly decreased GFP expression after 24 h and showed tendencies for this reduction at all other time points (Fig. 4F). Finally, we employed the endogenous SHANK3 expression of the HEK293T cells to analyze SHANK3 mRNA amount under experimental conditions in a non-neuronal system. Upon transfection of HEK293T cells with DNA or RNA ASOs, we found significantly decreased CT values after qRT-PCR analysis, indicating an increase in *SHANK3* mRNA. When converted into percentages, we found the ASOs identified to significantly increase *SHANK3* mRNA by about 8%–10% (Fig. 4H).

### Effect of 18nt RNA versus DNA ASOs in CTRL and PMDS hiPSCs after 72 h transfection

**FIG. 5.**
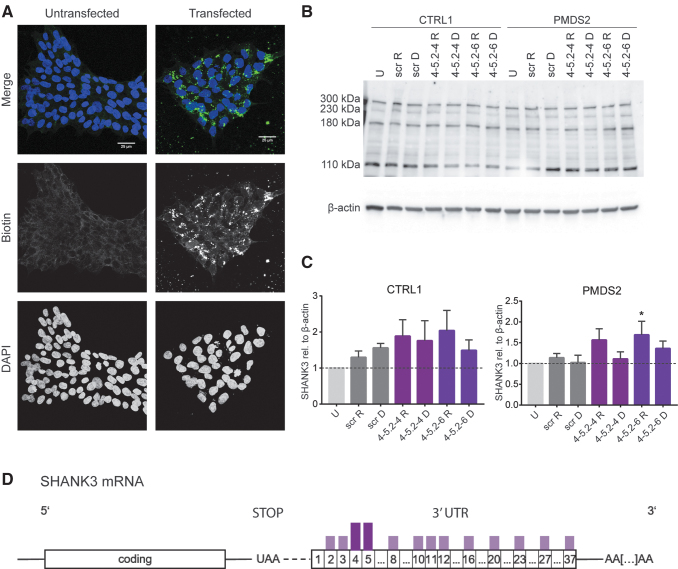
Effect of RNA and DNA ASOs (18mers) on SHANK3 levels in hiPSCs 72 h after transfection. **(A)** hiPSCs transfected for 72 h with the EV Shuttle Transfection Kit and stained for DAPI (*blue*) and biotin (*green*). Transfection efficiency was high in small colonies. **(B)** Western blot of transfected CTRL and PMDS hiPSCs. **(C)** SHANK3 levels as detected by analyzing the 300, 230, 180, and 110 kDa isoforms (*n* = 5, ANOVA, bars represent mean + SEM). **(D)** Schematic overview of the SHANK3 mRNA showing the 3′-UTR that has been divided into 50nt non-overlapping ASOs. ASOs that upregulated Shank3 protein expression are shown via a *purple bar* above their box. ASO 4 and ASO 5 were found to be most effective in the initial screen (*deep purple*) and ASO 4 and ASO 5 derived 18mers were subsequently characterized in this study (see also Table 1).

After confirming the effect of the DNA and RNA 18nt ASOs in HEK293T cells, we got back to the hiPSC lines to compare the potency after a longer transfection time. For this we established a hiPSC transfection method using the EV Shuttle Transfection Kit (EV = extracellular vesicles, including exosomes) for ASO delivery. This led to a reliable transfection and cell survival (Fig. 5A).

Transfected hiPSCs were collected after 72 h and protein expression was analyzed (Fig. 5B, C). We found no significant differences between untransfected and transfected CTRL hiPSCs; however, DNA 4-5.2-4 led to a 1.7-fold higher SHANK3 expression and RNA 4-5.2-4 a 1.9-fold increase (Fig. 5C). DNA 4-5.2-6 showed the least effect and increased detected SHANK3 1.5-fold whereas the RNA 4-5.2-6 yielded 2-fold the amount of SHANK3 (Fig. 5C). In PMDS hiPSCs, RNA 4-5.2-6 significantly elevated SHANK3 levels 1.7-fold whereas DNA 4-5.2-6 yielded a 1.4-fold increase in SHANK3 protein. DNA 2-4 showed no increase; however, RNA 4-5.2-4 increased SHANK3 protein levels by 1.6-fold (Fig. 5C). In summary, the effective ASOs identified are located in the very first part of the *SHANK3* 3′-UTR (Fig. 5D) and shorter 18nt ASOs have been identified from ASO 4 and ASO 5.

## Discussion

In this study we identified two 18nt ASOs that increase SHANK3 expression in controls and PMDS hiPSCs and hiPSC-derived motoneurons. In a step-wise procedure we first screened the complete sequence of the 3′-UTR of the human *SHANK3* gene by dissecting the UTR into 50nt long stretches leading to a first set of 36 ASOs. Those (as well as the 18nt ASOs derived from the effective ones) were then used to evaluate their enhancing effect on SHANK3 levels in hiPSCs of control and PMDS-patients.

ASOs are mainly used to reduce protein expression by steric hindrance or by activating cleavage by RNase H [25]. However, there are also studies describing the use of small RNAs to promote stability of the organism's RNA therefore leading to enhanced gene expression [42]. Screening for SHANK3 levels in control and PMDS-patient hiPSCs transfected with 50nt ASOs resulted in the identification of two clusters of ASOs to be effective in terms of upregulating properties, namely the ASO clusters 2 to 5 and 9 to 12. Interestingly, these ASO clusters are found in the first third of the 3′-UTR of the human *SHANK3* mRNA indicating that the target sequences that mediate an upregulation in SHANK3 expression are not equally distributed along the 3′-UTR (see also Supplementary Fig. S6).

Finally, we could identify effective 18nt ASOs that were able to significantly increase SHANK3 protein concentration in hiPSCs and hiPSC-derived motoneurons. With respect to western blotting it has to be considered that the calculated transfection efficiency was ∼10%–20%, meaning that the observed effects should be even more evident when reaching a higher transfection efficiency.

To further examine the target specificity of our ASOs, we designed vectors containing the human or mouse *SHANK3* 3′-UTR fused to GFP. By transfecting HEK293T cells with these constructs, we could observe whether the amount of expressed GFP varied upon transfection with the ASOs. Further, we were intrigued to see whether DNA and RNA ASOs would lead to different effects on SHANK3 expression. The detected increase in GFP protein amount confirmed the binding domains of the ASOs and supported their observed efficacy in a non-neuronal, SHANK3-coding-sequence independent manner. ASO 4-5.2-6 is laying in a conserved area of the human and the mouse 3′-UTR, ASO 4-5.2-4 includes a one nt change between both species. Still, we could observe effect of both ASOs on the GFP expression independent of which 3′-UTR had been transfected. By detecting endogenously expressed *SHANK3* mRNA levels in ASO-transfected HEK293T cells, we found that ASO 4-5.2-4 and ASO 4-5.2-6 increased mRNA levels significantly.

Li *et al*. previously reported the use of ASOs to increase and stabilize mRNA levels for the yield of higher protein levels [34]. We hypothesized that the *SHANK3* ASOs also have a stabilizing effect on *SHANK3* mRNA levels. However, we could not confirm this via transcription/translation inhibition since *SHANK3* has a half-life of >20 h and neither the HEK293T cells nor the iPSCs survived this extended period while administering a transcription inhibitor [43]. It has also to be considered that the identified ASOs might block the binding of microRNAs and by this prolong the half-life of the SHANK3 mRNA [35,36].

SHANK3 related ASD therapy using specific ASOs has not been described in literature so far. However, a lot of research has been done on the question whether disease-typical phenotypes can be reversed when SHANK3 levels are restored to physiological levels in the living animal. Mei *et al.* [12] managed to restore *Shank3* expression in a tamoxifen-inducible Cre-mouse model and they also found some behavioral aspects to be rescued, namely the repetitive grooming and social deficits. Shcheglovitov *et al.* [10] described that SHANK3 levels in human hiPSC-derived neurons from PMDS patients are significantly decreased. They could show that an increase of SHANK3 levels by transfection with a *SHANK3-GFP* construct or treatment with IGF-1 results in the restoration of almost all detected deficits.

Another recent, promising approach to restore SHANK3 levels is the treatment with Lithium [11,44]. SHANK3 increase after Lithium treatment led to a rescue of synaptic deficits *in vitro* and application to patients reversed behavioral phenotypes and catatonia. In conclusion, a rescue of SHANK3 levels appears to be a promising treatment of *SHANK3* associated ASD-like phenotypes [45,46]. In our study we investigated an ASO-based therapeutic approach that resulted in a SHANK3 protein upregulation *in vitro* of about 1.3–1.6 fold. Since SHANK3 expression in PMDS patients carrying a heterozygous SHANK3 deletion appears to be isoform dependent^11^ and reaches 50%–80% of control values, the ASO approach proposed here could well be of therapeutic value. Point mutations within the SHANK3 gene, however, would need to be analyzed with respect to a loss versus gain of function phenotype.

ASO based treatments have already been approved for SMA where Nusinersen is applied via intrathecal/spinal injection and significantly ameliorates the disease phenotype. Other ASOs have progressed to clinical trial stages—phase I and phase II—for treatment of other diseases such as Duchenne muscular dystrophy [47] or neurodegenerative diseases such as amyotrophic lateral sclerosis [48]. Limitations in ASO therapy are still the delivery to the nervous system and the stability/uptake. Up to now, a systemic delivery of ASOs is not possible owing to the blood brain barrier or the blood spinal cord barrier [23]. However, various delivery methods have been used to transport substances to the central nervous system, for example, direct infusion into the cerebrospinal fluid [46], intranasal delivery [49], or even intravitreal injection [50].

## Conclusion

We found that the 3′-UTR of human *SHANK3* mRNA harbors ASOs target sequences that enhance SHANK3 protein concentrations in hiPSCs and hiPSC-derived motoneurons. In further studies the *SHANK3* ASOs identified need to be tested not only for effectivity but also for pharmacokinetics, pharmacovigilance, and safety in an appropriate *in vivo* system. Also, the dosage of the ASOs needs to be carefully determined because SHANK3 levels should be restored only to physiological levels [3,51].

## Supplementary Material

Supplemental data

Supplemental data

Supplemental data

Supplemental data

Supplemental data

Supplemental data

Supplemental data

Supplemental data
