# Supplementary material for: Elevation of SHANK3 Levels by Antisense Oligonucleotides Directed Against the 3′-UTR of the Human SHANK3 mRNA
Source: Nucleic Acid Ther. 2023 Feb 1;33(1):58–71. doi: 10.1089/nat.2022.0048 (PMC9940809; doi:10.1089/nat.2022.0048)

**Supplementary Table 1: Overview over used hiPSC lines.** Depicted are the cell lines, their genotype, gender, diagnoses and clinical phenotypes (also used in the paper by 11).


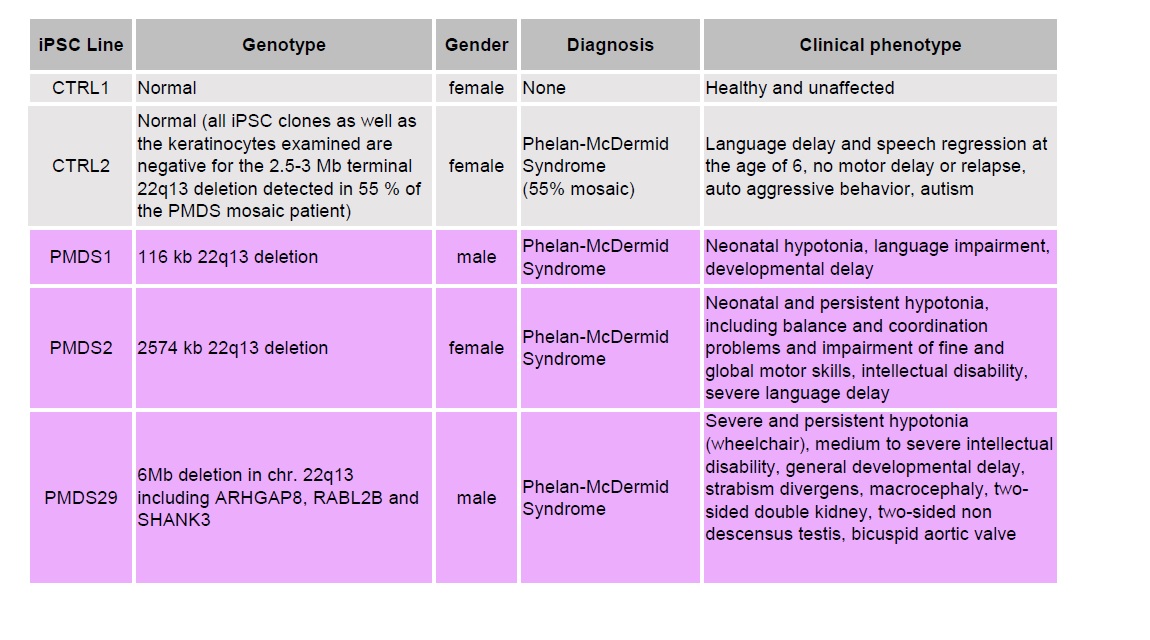

Supplement: Supplemental data [file Supp_TableS1.docx]
