# Supplementary material for: Elevation of SHANK3 Levels by Antisense Oligonucleotides Directed Against the 3′-UTR of the Human SHANK3 mRNA
Source: Nucleic Acid Ther. 2023 Feb 1;33(1):58–71. doi: 10.1089/nat.2022.0048 (PMC9940809; doi:10.1089/nat.2022.0048)

**Supplementary Figure 1: Western Blot results for all screened 50nt ASOs.** Blots for CTRL1 iPSCs transfected with ASOs2-37 and scrambled ASO **(A, C, E)** and for PMDS2 iPSCs transfected with the same ASOs **(B, D, F)**. The 110 kDa isoform was analyzed in all blots and β-actin was used for normalization.


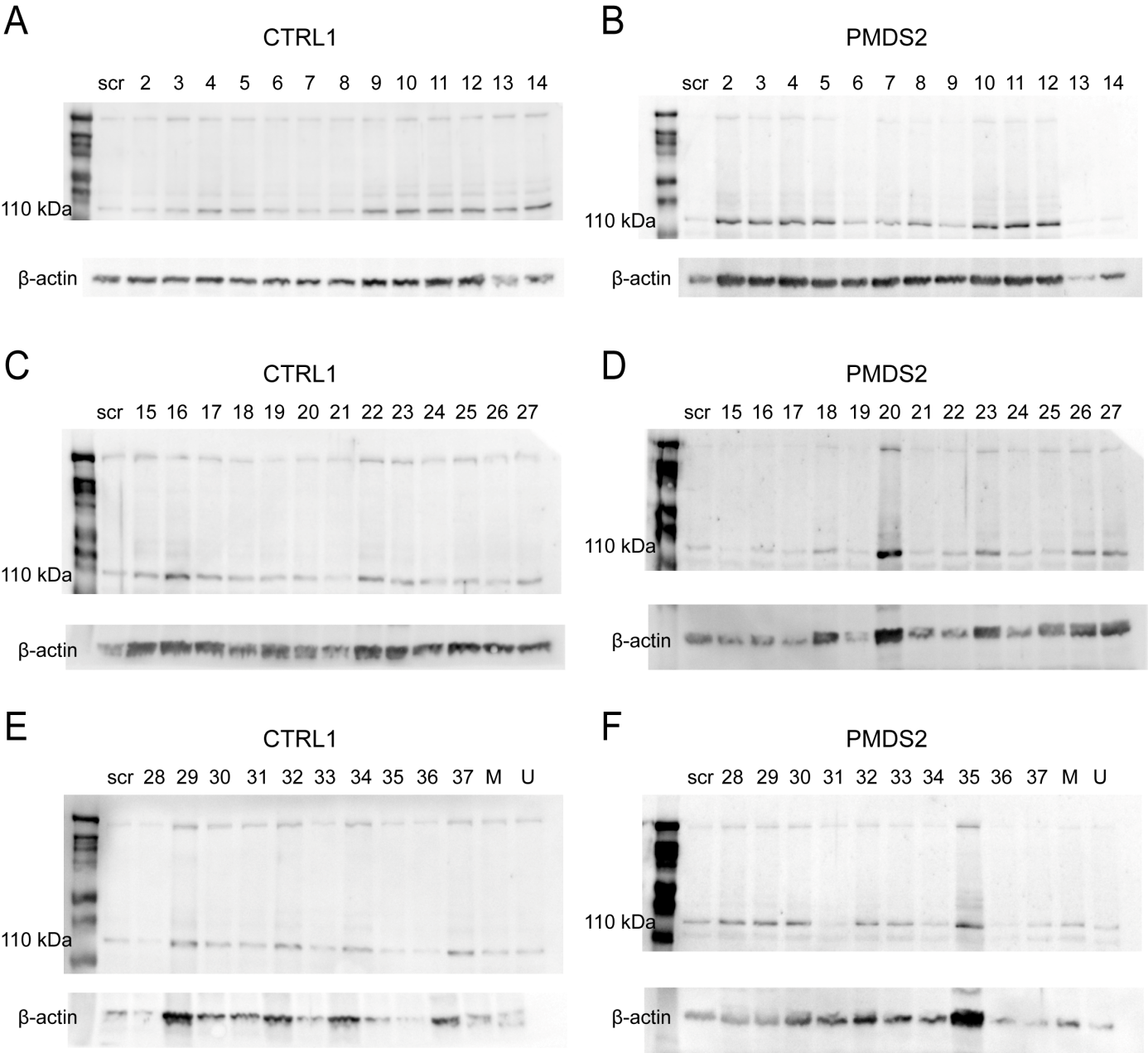

Supplement: Supplemental data [file Supp_FigS1.docx]
