# Supplementary material for: Elevation of SHANK3 Levels by Antisense Oligonucleotides Directed Against the 3′-UTR of the Human SHANK3 mRNA
Source: Nucleic Acid Ther. 2023 Feb 1;33(1):58–71. doi: 10.1089/nat.2022.0048 (PMC9940809; doi:10.1089/nat.2022.0048)
Supplement: Supplemental data [file Supp_TableS2.docx]

| Name | Sequence | %GC | Tm |
| --- | --- | --- | --- |
| scrambled | G*GCGCTCCGATGGTTCCAATGAACA*CGTCGCCCACGCCGGATGAGCTTC*T |  |  |
| ASO1 | Could not be synthesized |  |  |
| ASO2 | T*GGCCCGGGCGCCGTCGTAAGGGGC*AGGCCGAGCCCGCGGCCCGGGGTG*G | 84 | >75 |
| ASO3 | C*AGGGCGAGTCTCCGAGCAACAGCA*AACAGGACGATTCATGCAACATTC*C | 54 | >75 |
| ASO4 | G*ACTGTGCGCTGGGTGGGCTGGGCG*GCCGGTGAGGGCACTAAGCAATGT*A | 66 | >75 |
| ASO5 | C*CCCACCCCCGGCACCTCCCGCCCC*AGCCTCCCTGGTCCACGCCCTTCC*T | 78 | >75 |
| ASO6 | C*TGGGAGAATGGCCACCAGGAGCTG*CGAAGGAGGTGGTCACGCTAGGGC*A | 64 | >75 |
| ASO7 | C*GAGTCACCAAGGCGGGTCCTGACC*TCGCATGCTGGACTAGGTTCCCCC*T | 64 | >75 |
| ASO8 | G*GGGGGCTCCTTGGCCCCCATCGAG*AATCCCAATGTCTCCCCCCTCCCC*C | 70 | >75 |
| ASO9 | T*TGTTCCTTTTCTCGTTCCAAATAT*AGAGTGGATTAAAATATGCAAAAC*A | 30 | 68.6 |
| ASO10 | C*CAGTGGAAGTAGAAGGGAGGGGAG*AGGAAACTATTACGGACAGAGATA*T | 48 | >75 |
| ASO11 | G*CAGTGGCAGTGAGGGGCCGGAGAT*GGAAGACTGGGCAGCTGCAGTGGG*A | 66 | >75 |
| ASO12 | C*CTGGCCAACCCCAGACCAGCTGGA*GCGTCCCCTGCCCCGTTGTGGGGT*G | 72 | >75 |
| ASO13 | C*TCAGCGAGGCGAGGGGCCGAGCTG*GGGCCCCAGGGCGGGCCACTAGGG*C | 80 | >75 |
| ASO14 | G*CCCCTCCCACCACGAGCAGCACCT*GAAGGGTCGGTGGGGCACACTAGA*G | 68 | >75 |
| ASO15 | C*TCCCAGGCCGGCTGGTCCCGCGGG*TGCACAGCAGGACCCGCGGCCTGC*C | 80 | >75 |
| ASO16 | G*GCTGCGGTGGAGCGGCAGGGCCCT*GCCCTCATCCCCCCCGGCCGATGG*T | 78 | >75 |
| ASO17 | A*TGGGGACCAAGCTAGCCCCCTCCT*TGGGGAGAGACCCTGTGAGGAAGA*T | 60 | >75 |
| ASO18 | A*CTTTGGGGTCCAAGGCTGGGAGGC*TTCTCTGCTGTCGTTGCCCAAGAG*C | 60 | >75 |
| ASO19 | T*GCTCAGACGCACCCAGTTTCACCC*ACACACATAAAGGGCAGGACAGGA*G | 56 | >75 |
| ASO20 | A*GCGGCCCCTGGTGGCCACTCAGTA*ATCAGGCACACACACGGCTCCCAC*G | 66 | >75 |
| ASO21 | A*CCCCACGCATGCACACGGTGCACG*CCTCCACGGCCCCGCGCTAGTCCA*G | 72 | >75 |
| ASO22 | A*TGGCCTCTGACTTTCTTTGGAAGA*GGAGACAGGGTGCTCTCACAGGTA*C | 52 | >75 |
| ASO23 | T*GAGGCTCTGGAAGGACAGGCTGGG*CAAACAGCTGGACCCAGGGTGCAG*G | 64 | >75 |
| ASO24 | G*GGCCTCCCCAAGCAGCAGGGATTC*ACCAGGGAACCCCGCTCAGGCTGG*G | 70 | >75 |
| ASO25 | T*GATGCCCTCAGAAGCCCAAGGTGG*GGGCGCTGCCTCCAAGGGGCCCTT*G | 68 | >75 |
| ASO26 | C*TTTGACTCTCCCCAGGGCCTGTGG*TGAACTGACTCTAGGGGTCCCCCT*A | 60 | >75 |
| ASO27 | T*CGCTGAGTGTGAGGAGTCACAGTG*TGGGGGGCTGGGCACCCTCGGGGG*T | 68 | >75 |
| ASO28 | T*CCAAACCCTAGGTTTAAAAACGTC*CCAGGGCCCCCCACCCCACAGGTC*A | 58 | >75 |
| ASO29 | G*AAATATAAACAGAAACTTGTGAGT*GACGTGGATGGAGCTTAGTCCAGA*C | 42 | 73.5 |
| ASO30 | T*TGTGAAAACTTCTGTTTTCTTTTT*TTTTTTATTTTATTAAAAAAAGCT*A | 16 | 62.9 |
| ASO31 | T*GGGCCAGGGCGGGGCGGGCAGGCA*CAGACCGGCGTGCCAGGCCCCTGG*G | 82 | >75 |
| ASO32 | T*TGGCGGAAGGATGAGTGGGTGTGA*CTCTGTGCCCAGGGAGTGGGGCCG*G | 66 | >75 |
| ASO33 | T*GTGTATGTGGCAGTCTGTTACAGT*GACTGCTGCTGTGTGACCTGGACT*G | 52 | >75 |
| ASO34 | A*AAACCCAAATTGAACGGAACCAAA*ACCCACAGGTGAGTGTGAGACCGA*G | 48 | >75 |
| ASO35 | A*TGGAACTCCATACAAAAGGAGGTG*AAGCGGAACTGACCCTGTAAAGTT*A | 44 | 74.3 |
| ASO36 | C*GTCAGGGTCAGGAGGCCTCAGGAC*TGGAGCAGGGGGTGAAACCCCCCG*G | 70 | >75 |
| ASO37 | A*TCAATAATAATAATATAAGAAACA*TAGATCTCTGTGGGGCGTATCACA*A | 30 | 68.6 |
| scr18 | G*CCCACGCC*GGATGAGC*T | 72.2 | 62.8 |
| ASO 4-5.1-1 | C*CCCACCCC*CGGCACCT*C | 83.3 | 67.4 |
| ASO 4-5.1-2 | C*CGCCCCAG*CCTCCCTG*G | 83.3 | 67.4 |
| ASO 4-5.1-3 | T*CCACGCCC*TTCCTGAC*T | 61.1 | 58.2 |
| ASO 4-5.1-4 | G*TGCGCTGG*GTGGGCTG*G | 77.8 | 65.1 |
| ASO 4-5.1-5 | G*CGGCCGGT*GAGGGCAC*T | 77.8 | 65.1 |
| ASO 4-5.1-6 | A*AGCAATGT*ACAGGGCG*A | 50 | 53.7 |
| ASO 4-5.2-1 | C*TAGGGCAC*CCCACCCC*C | 77.8 | 65.1 |
| ASO 4-5.2-2 | G*GCACCTCC*CGCCCCAG*C | 83.3 | 67.4 |
| ASO 4-5.2-3 | C*TCCCTGGT*CCACGCCC*T | 72.2 | 62.8 |
| ASO 4-5.2-4 | T*CCTGACTG*TGCGCTGG*G | 66.7 | 60.5 |
| ASO 4-5.2-5 | T*GGGCTGGG*CGGCCGGT*G | 83.3 | 67.4 |
| ASO 4-5.2-6 | A*GGGCACTA*AGCAATGT*A | 44.4 | 51.4 |
| RNA scr* | <G*C*C*C*A*C*G*C*C*G*G*A*T*G*A*G*C*T> | 50 | 46 |
| RNA ASO 2-4* | <T*C*C*T*G*A*C*T*G*T*G*C*G*C*T*G*G*G> | 38.9 | 40 |
| RNA ASO 2-6* | <A*G*G*G*C*A*C*T*A*A*G*C*A*A*T*G*T*A> | 27.8 | 40 |
| DNA scr* | G*C*C*C*A*C*G*C*C*G*G*A*T*G*A*G*C*T | 50 | 46 |
| DNA ASO 2-4* | T*C*C*T*G*A*C*T*G*T*G*C*G*C*T*G*G*G | 38.9 | 40 |
| DNA ASO 2-6* | A*G*G*G*C*A*C*T*A*A*G*C*A*A*T*G*T*A | 27.8 | 40 |
|  | *These ASOs were additionally synthesised carrying a 6-FAM tag at the 5’ end |  |  |
