# Supplementary figures and images for: Elevation of SHANK3 Levels by Antisense Oligonucleotides Directed Against the 3′-UTR of the Human SHANK3 mRNA
Source: Nucleic Acid Ther. 2023 Feb 1;33(1):58–71. doi: 10.1089/nat.2022.0048 (PMC9940809; doi:10.1089/nat.2022.0048)

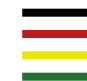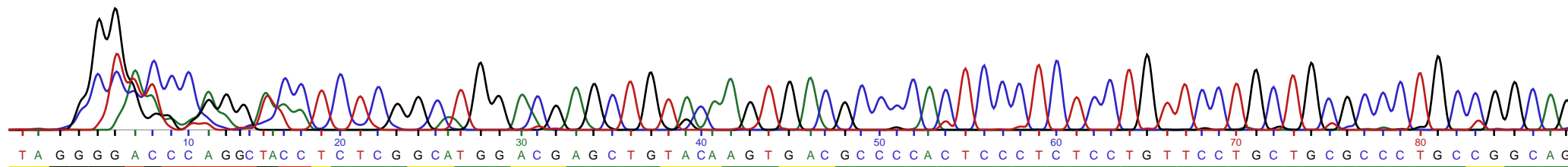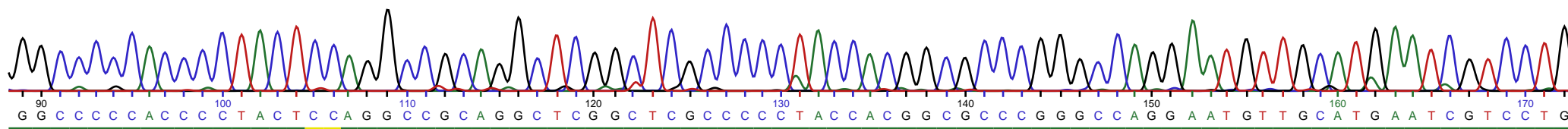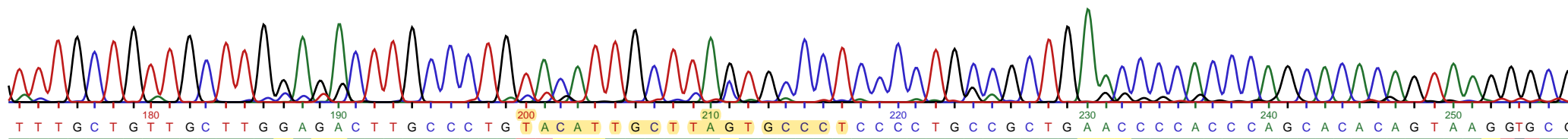

## AON 2-6

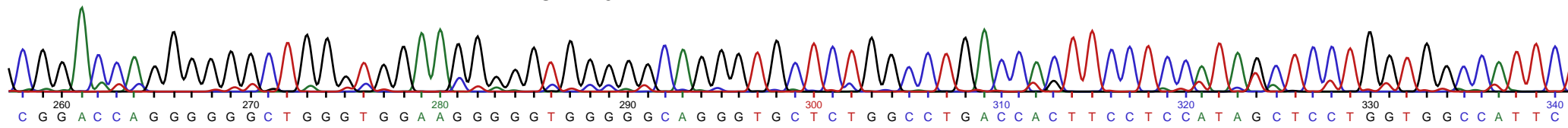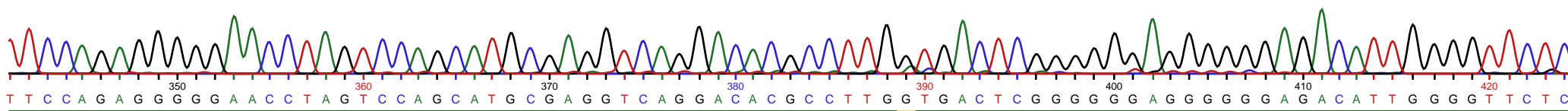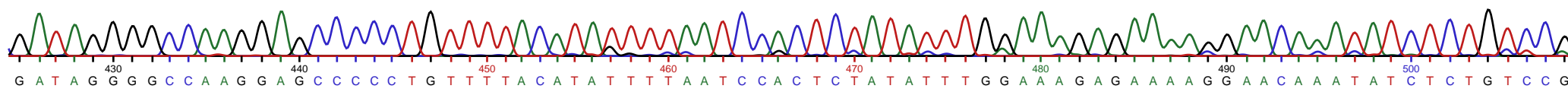

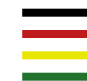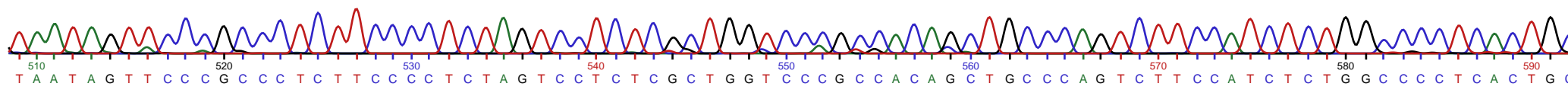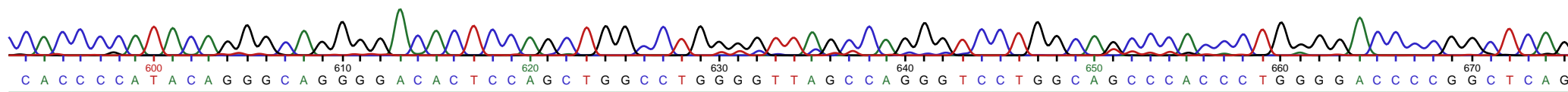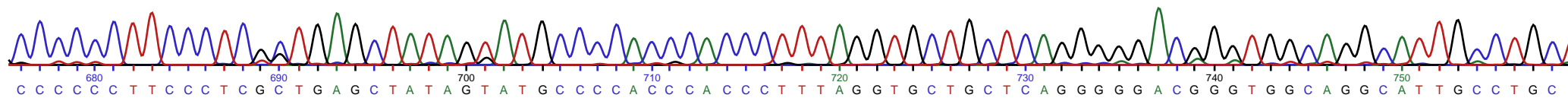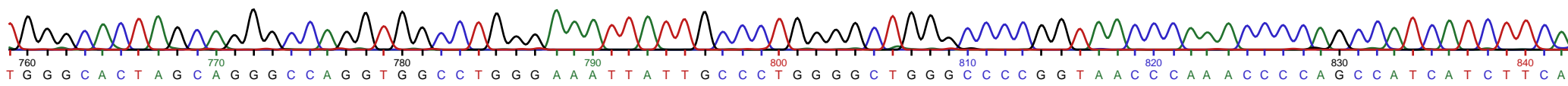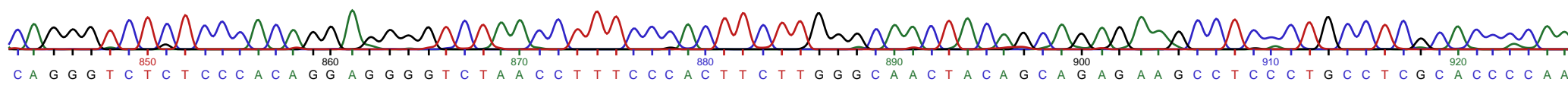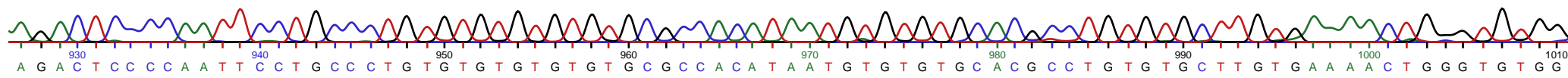

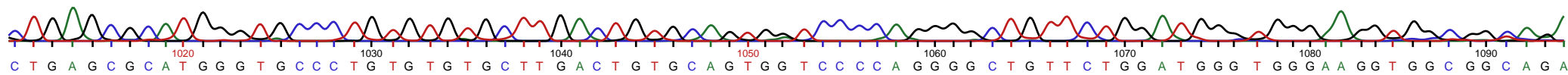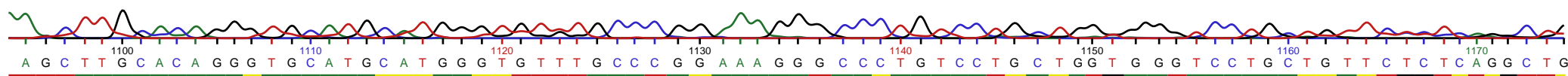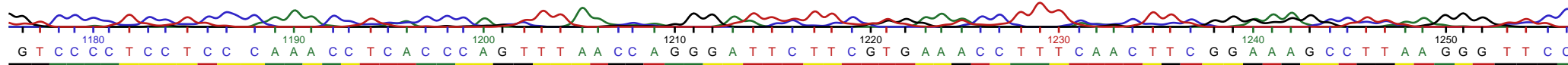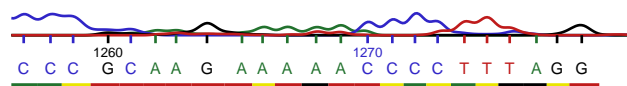

Supplement: Supplemental data [file Supp_figS5.pdf]

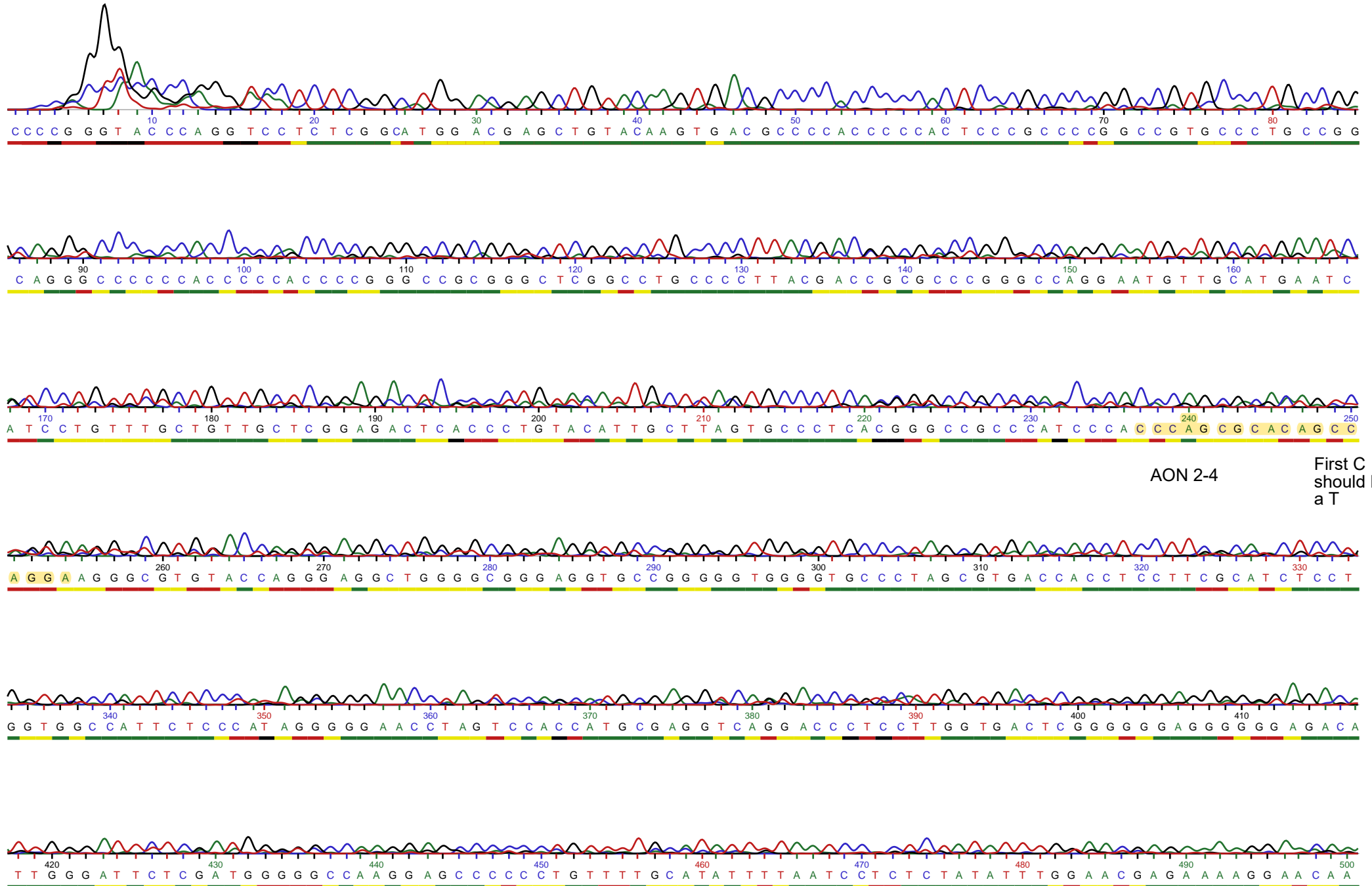

AON 2-4

First C  
should be  
a T

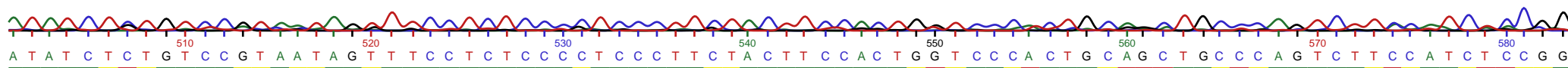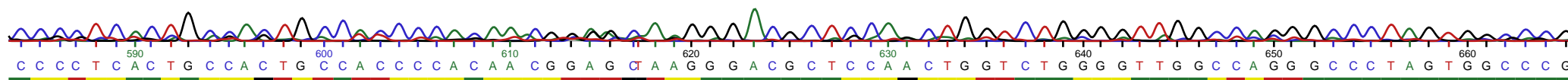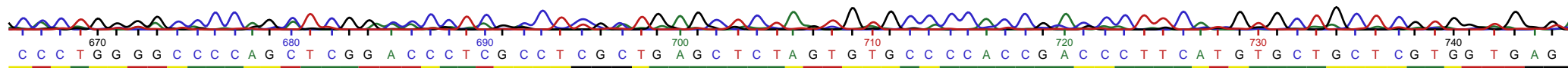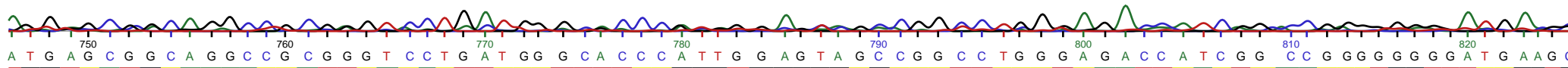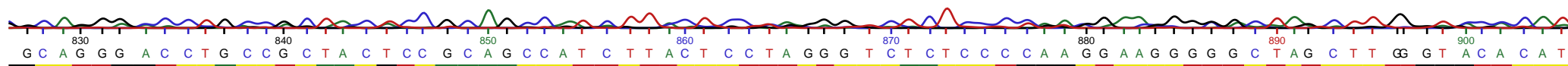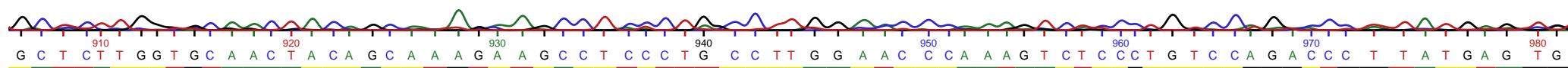

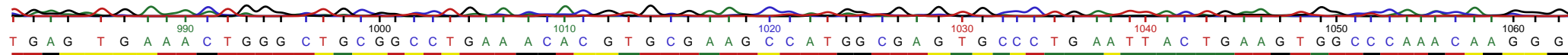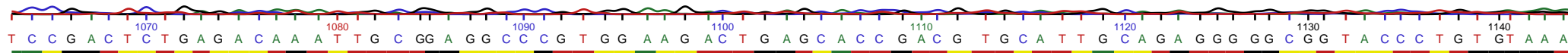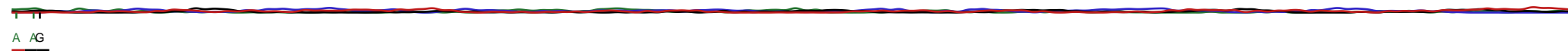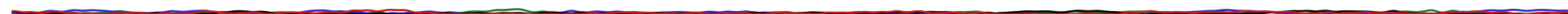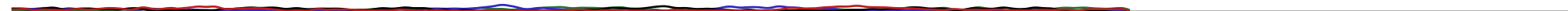

Supplement: Supplemental data [file Supp_figS3.pdf]
