# Supplementary material for: Elevation of SHANK3 Levels by Antisense Oligonucleotides Directed Against the 3′-UTR of the Human SHANK3 mRNA
Source: Nucleic Acid Ther. 2023 Feb 1;33(1):58–71. doi: 10.1089/nat.2022.0048 (PMC9940809; doi:10.1089/nat.2022.0048)

**Supplementary** **Figure** **4:** **Vector card of the plasmid containing the GFP - murine *Shank3* 3’UTR sequence.** Promotor, restriction sites, transcription activation site and more are depicted in the graph.


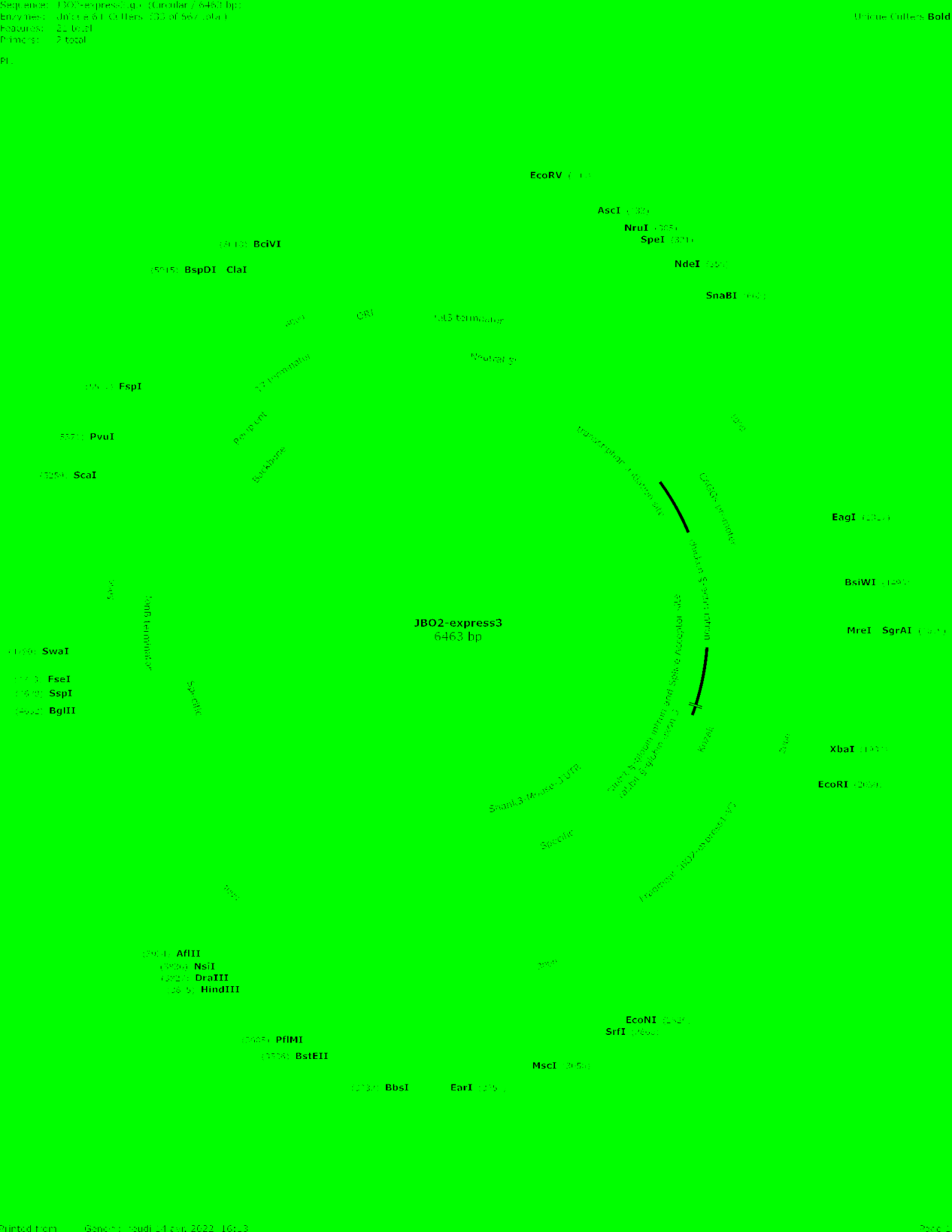

Supplement: Supplemental data [file Supp_FigS4.docx]
