# Supplementary material for: Elevation of SHANK3 Levels by Antisense Oligonucleotides Directed Against the 3′-UTR of the Human SHANK3 mRNA
Source: Nucleic Acid Ther. 2023 Feb 1;33(1):58–71. doi: 10.1089/nat.2022.0048 (PMC9940809; doi:10.1089/nat.2022.0048)

**Supplementary** **Figure** **6: Secondary structure of the human *SHANK3* 3’UTR.** Enlarged window shows the region of the 3’UTR where ASOs 4 and 5 are located. RNAfold was used to generate secondary structure: <http://rna.tbi.univie.ac.at//cgi-bin/RNAWebSuite/RNAfold.cgi?PAGE=3&ID=F64cNC9CBw> (02.09.2022, 10 am)


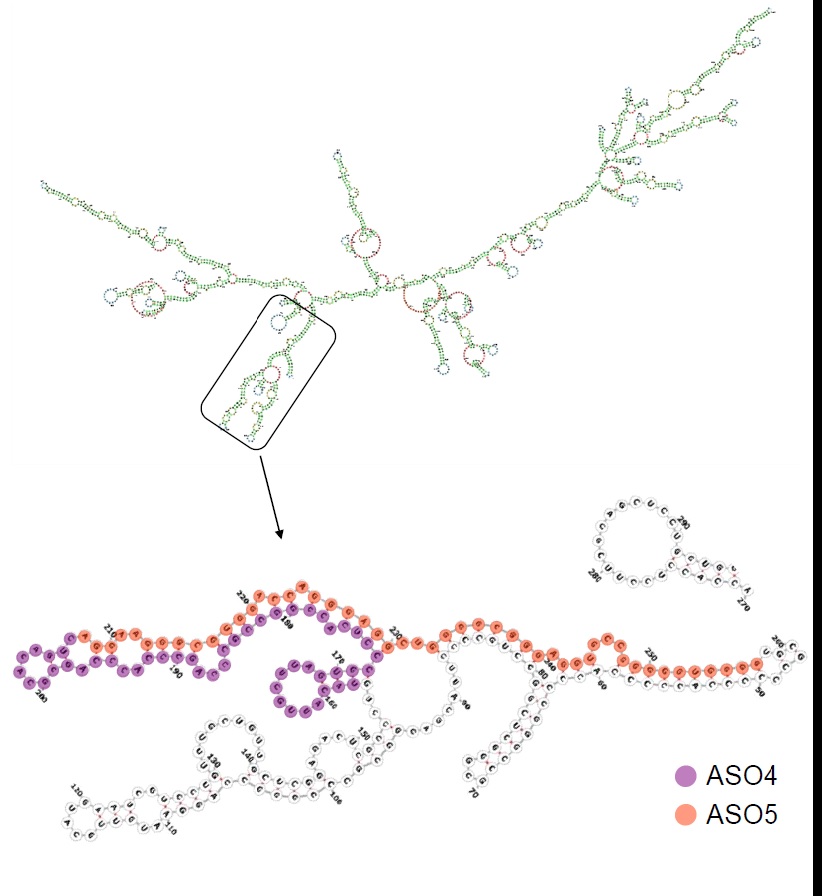

Supplement: Supplemental data [file Supp_FigS6.docx]
